# Delayed effect of bifrontal transcranial direct current stimulation in patients with treatment-resistant depression: a pilot study

**DOI:** 10.1186/s12888-019-2119-2

**Published:** 2019-06-11

**Authors:** Min-Shan Li, Xiang-Dong Du, Hsiao-Chi Chu, Yen-Ying Liao, Wen Pan, Zhe Li, Galen Chin-Lun Hung

**Affiliations:** 1 Blossom Clinic of Psychosomatic Medicine, Taipei, Taiwan; 2 Suzhou Guangji Hospital, Suzhou, China; 3 Affiliated Guangji Hospital of Soochow University, Suzhou, China; 4 Department of Public Health, School of Medicine, National Yang-Ming University, Taipei, Taiwan; 5 Taipei City Psychiatric Center, Taipei City Hospital, Taipei, Taiwan

**Keywords:** Transcranial direct-current stimulation, Treatment-resistant depression, Cognitive ability

## Abstract

**Background:**

Transcranial direct current stimulation (tDCS) is a non-invasive brain stimulation technique, which has yielded promising results in treating major depressive disorder. However, its effect on treatment-resistant depression remains to be determined. Meanwhile, as an emerging treatment option, patients’ acceptability of tDCS is worthy of attention.

**Methods:**

This pilot study enrolled 18 patients (wome*n* = 13) with treatment-resistant unipolar (*n* = 13) or bipolar (*n* = 5) depression. Twelve sessions of tDCS were administered with anode over F3 and cathode over F4. Each session delivered a current of 2 mA for 30 min per ten working days, and at the 4th and 6th week. Severity of depression was determined by Montgomery–Åsberg Depression Rating Scale (MADRS); cognitive performance was assessed by a computerized battery.

**Results:**

Scores of MADRS at baseline (29.6, SD = 9.7) decreased significantly to 22.9 (11.7) (*p* = 0.03) at 6 weeks and 21.5 (10.3) (*p* = 0.01) at 8 weeks. Six (33.3%) participants were therapeutically responsive to tDCS. MADRS scores of responders were significantly lower than those of non-responders at the 6th and 8th week. Regarding change of cognitive performance, improved accuracy of paired association (*p* = 0.017) and social cognition (*p* = 0.047) was observed at the 8th week. Overall, tDCS was perceived as safe and tolerable. For the majority of patients, it is preferred than pharmacotherapy and psychotherapy.

**Conclusions:**

TDCS can be a desirable option for treatment-resistant depression, however, its efficacy may be delayed; identifying predictors of therapeutic response may achieve a more targeted application. Larger controlled studies with optimized montages and sufficient periods of observation are warranted.

**Trial registration:**

This trial has been registered at the Chinese Clinical Trial Registry (ChiCTR-INR-16008179).

## Background

Major depressive disorder (MDD) is a highly prevalent mental illness associated with substantial personal impairments and societal costs [1]. Although progress has been made in the pharmacological and psychotherapeutic intervention of MDD, there are still up to 50% of patients with poor response to multiple trials of antidepressants, defined as treatment-resistant depression (TRD) [2–5]. Patients with TRD have lower quality of life. They account for more frequent medical visits and higher health care costs [6].

Empirical pharmacotherapy of TRD includes augmentation with lithium or second generation antipsychotics, often with suboptimal efficacy and poor tolerance [7]. Electroconvulsive therapy (ECT) remains as an efficient treatment for TRD, however, its application is limited due to risk of anesthesia and cognitive side effects [7–9]. Indeed, current treatment for TRD is still far from satisfactory; there is an urgent need for novel therapeutics. Recently, non-invasive brain stimulation has emerged as a promising candidate. Repetitive transcranial magnetic stimulation (rTMS) has been approved by the US Food and Drug Administration for treating TRD [10]; interests in transcranial direct current stimulation (tDCS) is growing following its demonstrated efficacy on MDD.

TDCS, a non-convulsive brain stimulation technique involves injecting a low-amplitude (generally 1-2 mA), direct electric current flows from the anode to the cathode in cerebral cortex by using two surface scalp electrodes [11, 12], which alters the membrane potentials of neurons and changes the rate of spontaneous depolarization [13–15]. The anode area becomes hypo-polarized and the cathode area becomes hyper-polarized. One well-known hypothesis of depression is hypoactivity in left dorsolateral prefrontal cortex (DLPFC) leading to psychomotor retardation and executive dysfunction [16, 17]. Researchers suppose that tDCS anodal stimulation over left DLPFC increases its cortical activity, which would lead to improvement of depression. In recent years, multiple randomized studies have confirmed the antidepressant effect of tDCS among patients with MDD [18–23]. Rigonatti et al. published that tDCS had equal but faster antidepressant effects in comparison with fluoxetine [24]. Brunoni and colleagues demonstrated that effects of tDCS plus antidepressants can be synergistic [25].

Given the encouraging findings of tDCS in MDD, several pilot studies have explored its effect in TRD patients, with mixed results. In a randomized controlled study applying 10 sessions of tDCS, there were no significant differences of depressive symptoms between active and sham groups after 4 weeks [26]. Blumberger and his team extended that a 15-session tDCS was not efficacious in TRD [27]. In another controlled study, tDCS efficacy on psychomotor and neuropsychological functioning in TRD is limited [28]. On the contrary, the promising result from Dell’Osso el al. revealed that tDCS administered twice a day for 5 consecutive days help reduce depressive symptoms, particularly melancholic features [29]. Another encouraging study conducted by Ferrucci applied the identical tDCS protocol among hospitalized patients with severe MDD; improvement was observed on day 5 after ten tDCS sessions and persisted to the end of 5 weeks [30].

It has been observed that the effect of brain stimulation can be delayed [31], with its effect manifesting beyond treatment periods. However, existing research often completed follow-up at the end of treatment. The majority of studies examined the effect of tDCS on depressive symptomatology, few have comprehensively assessed cognitive performance, a crucial determinant of functional recovery. Moreover, as a novel treatment modality, the acceptability of tDCS needs to be further established. Thus, in the present study, we examined, at the 8th week, the antidepressant and cognitive effects of a 6-week tDCS treatment for TRD, identified potential predictors for treatment responsiveness, and elaborated the subjective experiences receiving tDCS.

## Methods

### Study participants

This pilot study recruited 18 patients (wome*n* = 13) meeting Diagnostic and Statistical Manual of Mental Disorders-5 (DSM-5) criteria for unipolar (n = 13) or bipolar (*n* = 5) depression at Taipei City Psychiatric Center, Taipei City Hospital. In unipolar depression patients, current depressive episode needed to be treatment-resistant, which was defined as failure to respond to 2 adequate trials of pharmacotherapy. The definition of treatment-resistant bipolar depression has not been established yet [32]. We followed Sachs’s definition of treatment-resistant bipolar depression: non-remission despite two adequate trials of standard antidepressant agents, with or without augmentation strategies [33]. Regimen of psychotropic needed to be fixed at least 4 weeks prior to enrollment, and maintain unchanged throughout the study. Participants must score over 20 on the Montgomery–Åsberg Depression Rating Scale (MADRS). Those with metal implants, intracranial lesions, cerebrovascular or cardiovascular diseases and pregnancy were excluded. Additionally, participants receiving DSM-5 diagnosis of schizophrenia and substance use disorder were excluded, as well as those failing to respond to previous ECT. This study conformed with the Declaration of Helsinki and received proper Institutional Review Board approval (TCHIRB-10409114). Written informed consent was obtained from each participant prior to study initiation. This trial has been registered at the Chinese Clinical Trial Registry (ChiCTR-INR-16008179).

### Transcranial direct-current stimulation

Direct current was generated by a constant-current stimulator (Starstim tCS, manufactured by Neuroelectrics, Barcelona). tDCS were administered with anode over F3 (International 10/20 System for EEG Electrodes) and cathode over F4. The size of conductive electrodes was about 35cm^2^. Each session delivered a direct current of 2 mA for 30 min. A total of 12 sessions were administrated, patients received daily tDCS administration for 10 days of the first 2 weeks and followed by a single tDCS administration on the 4th and 6th week [25]. The single booster stimulation on the 4th and 6th week was implemented due to our previous experiences that effects of tDCS may diminish after a 2-week stimulation protocol. After 12 sessions of tDCS administration, the patients received an intervention-free follow-up observation at the 8th week.

### Psychiatric assessment

Severity of depression was measured by the Montgomery–Åsberg Depression Rating Scale (MADRS), a 10-item instrument tapping into the symptomatology of depression. MADRS was measured at baseline, the 2nd, 4th, 6th, and 8th week. To ensure the reliability, all the assessments were carried out by one senior psychiatrist (G.C.H). Response to tDCS was defined by a > 50% decrease of MADRS scores. For each assessment, Beck Depression Inventory (BDI) was self-administered as a complimentary outcome.

### Cognitive assessment

Cognitive performance was assessed by a computerized battery, COGSTATE, which included 11 tasks examining domains of attention, working memory, verbal and visual memory, social cognition and executive function. Performance was evaluated either by accuracy rate, number of errors or reaction time.

### Subjective experiences of tDCS

Structured interview was conducted to elicit: 1) participants’ understanding of tDCS; 2) adverse effects of tDCS; 3) perceived benefits of tDCS on mood and cognition, and 4) preference of tDCS in comparison with pharmacotherapy and psychotherapy. A research assistant (L.Y.Y.) uninvolved in the study procedure conducted the interview independently.

### Statistical analysis

Paired t-test was used to examine changes of MADRS and BDI over 8 weeks, as compared to scores at baseline. As for cognitive outcomes, paired t-test was used to compare the reaction time and accuracy of a given task at baseline and at 8 weeks. With an intention to identify predictors for tDCS response, we used Fisher’s exact test (for categorical variables) and unpaired t-test (for continuous variables) to compare the demographic and clinical characteristics of tDCS responders and non-responders. Compared to baseline, differences between responders and non-responders on change of MADRS, BDI and cognitive scores at 2, 4, 6, and 8 weeks were examined by paired t-tests. Effect size (Cohen’s d) was then calculated.

## Results

### Participants

We enrolled 18 patients (wome*n* = 13) with treatment-resistant unipolar (n = 13) or bipolar (*n* = 5) depression. Average age was 44.6 (SD = 14.2) years, with onset of depression at 29.8 (15.6) years. Twelve (66.7%) participants had visited psychiatric ER, and 10 (55.6%) had attempted suicide, indicating a more severe course of depression. Eight (44.4%) participants reported family history of psychiatric disorders. The majority of participants received antidepressants and benzodiazepines. Notably, half of them were prescribed with antipsychotics (Table 1).

**Table 1:** Comparison of clinical characteristics and severity of depression in all participants, responders and non-responders

	All participants		Responders *N* = 6		Non-responders *N* = 12		*P*-value
Age (years)	44.6	14.2	47.7	12.5	43	15.3	.527
Sex							1.00
Male	5	27.8%	2	33.3%	3	25%	
Female	13	72.2%	4	66.7%	9	75%	
Diagnosis							1.00
MDD	13	72%	4	66.7%	9	75%	
Bipolar	5	28%	2	33.3%	3	25%	
Onset age (years)	29.8	15.6	29	14.6	30.2	16.8	.887
Ever received ECT							1.00
No	16	88.9%	5	83.3%	11	91.7%	
Yes	2	11.1%	1	16.7%	1	8.3%	
Every visited ER for psychiatric emergency							.344
No	6	33.3%	3	50%	3	33.3%	
Yes	12	66.7%	3	50%	9	66.7%	
History of suicide attempt							.321
No	8	44.4%	4	66.7%	4	33.3%	
Yes	10	55.6%	2	33.3%	8	66.7%	
Family history of psychiatric disorders							.638
No	10	55.6%	4	66.7%	6	50%	
Yes	8	44.4%	2	33.3%	6	50%	
With antidepressants							1.00
No	2	11.1%	1	16.7%	1	8.3%	
Yes	16	88.9%	5	83.3%	11	91.7%	
With antipsychotics							1.00
No	9	50%	3	50%	6	50%	
Yes	9	50%	3	50%	6	50%	
With mood stabilizers							1.00
No	14	77.8%	5	83.3%	9	75%	
Yes	4	22.2%	1	16.7%	3	25%	
With benzodiazepines							1.00
No	1	5.6%	0	0%	1	83.3%	
Yes	17	94.4%	6	100%	11	91.7%	
MADRS at baseline	29.61	9.71	30.83	15.09	29	6.41	.718
BDI at baseline	34.56	13.05	31.83	15.74	35.92	12.02	.548

*MADRS* Montgomery–Åsberg Depression Rating Scale, *BDI* Beck Depression Inventory; numbers in the table are either means with standard deviation or counts with percentage

### Depressive symptoms

**Fig. 1:**
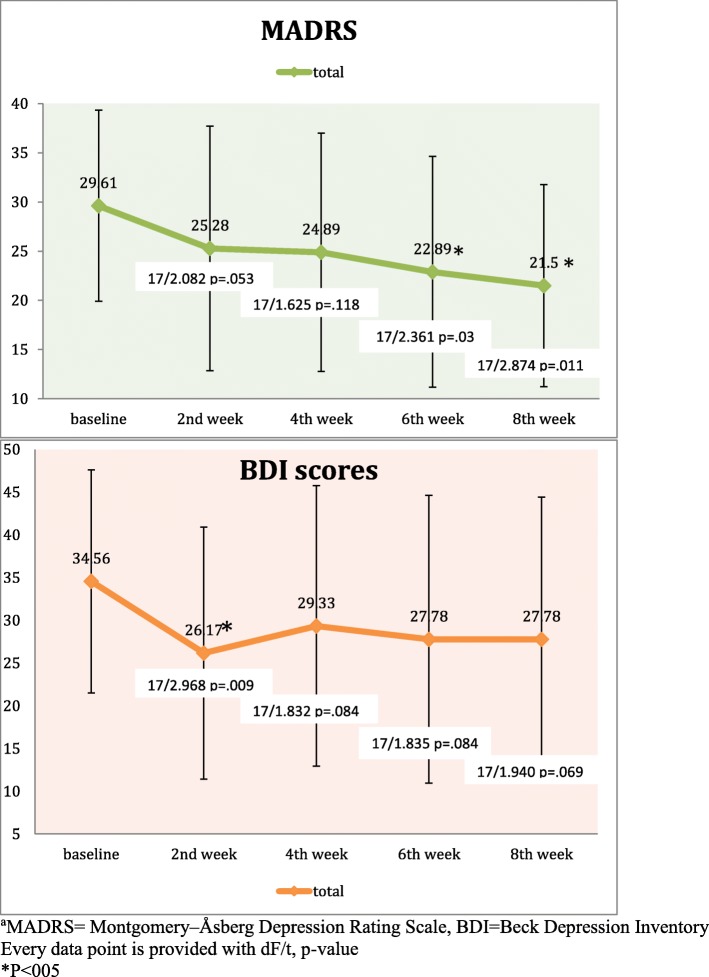
Change of depressive symptoms over 8 weeks

**Fig. 2:**
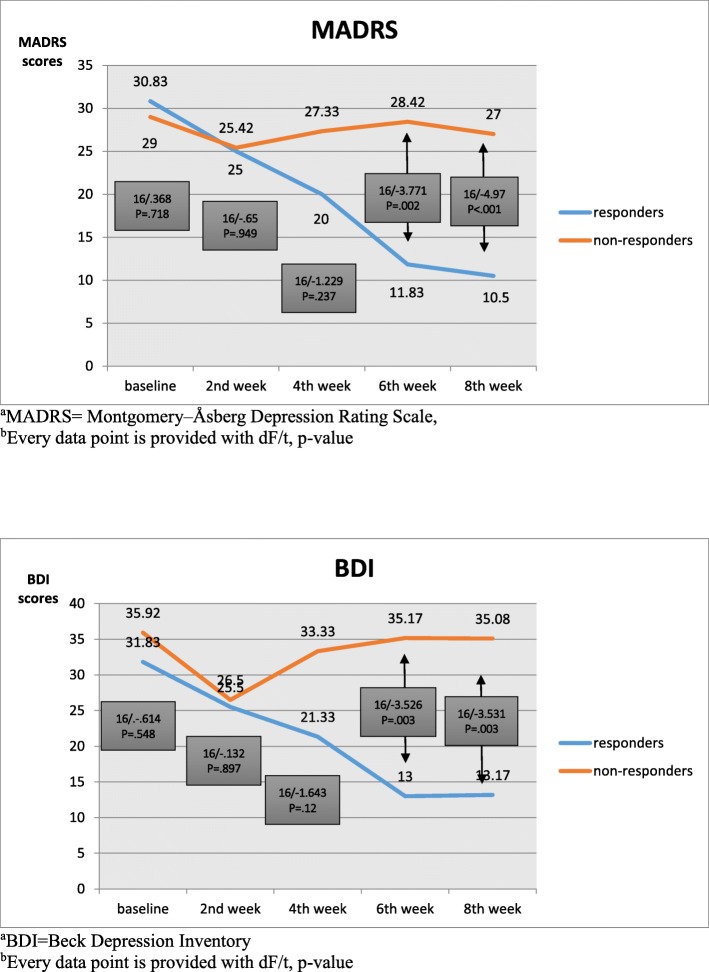
Comparison of change of depressive symptoms in responders and non-responders

In the current sample, scores of MADRS at baseline (29.6, SD = 9.7) showed a significant decrease at week 6 (t = − 2.361; *p* = .03) and week 8 (t = 2.874; *p* = 0.011). As for BDI, a significant change from baseline was observed only at week 2 (t = 2.968; *p* = .009) (Fig. 1). Six (33.3%) participants were therapeutically responsive to tDCS. Paired t-tests revealed a more pronounced decrease in MADRS scores of responders than non-responders at weeks 6 (t = − 3.771, *p* = .002, Cohen’s d = 1.71) and 8 (t = − 4.97, *p* < .001, Cohen’s d = 2.44). Similarly, paired t-tests revealed a more pronounced decrease in BDI scores of responders than non-responders at weeks 6 (t = − 3.526, *p* = .003, Cohen’s d = 1.92) and 8 (t = − 3.531, *p* = .003, Cohen’s d = 1.92) (Fig. 2). Treatment responsiveness was not predicted by any demographic or clinical characteristics and cognitive performance at baseline (Table 1).

### Cognitive performance

Among 11 cognitive tasks administered, improved accuracy of one task regarding visual learning (*p* = 0.017) and another regarding social cognition (*p* = 0.047) was observed at the 8th week (Table 2). Change of cognitive performances was similar in responders and non-responders (data not shown).

**Table 2:** Comparison of performances regarding tasks in Cogstate at baseline and at 8 weeks

Test item	baseline	8 weeks	t/dF	p
Shopping list				
Time Spent (sec)	216,301	227,146.36	−8.35/13	.419
Accuracy (sin^−1^)	.920322	.884031	1.033/13	.321
Detection Task				
Reaction Time (log)	2.664721	2.694657	−1.332/13	.206
Accuracy (sin^−1^)	1.492700	1.490980	.039/13	.969
Identification Task				
Reaction Time (log)	2.784243	2.792496	−.302/13	.767
Accuracy (sin^− 1^)	1.336756	1.356455	−.376/13	.713
One Card Learning Task				
Reaction Time (log)	3.035103	3.052699	−.601/13	.558
Accuracy (sin^−1^)	.921514	.899249	.937/13	.366
One Back Working Memory Task				
Reaction Time (log)	2.954759	2.973666	−.454/13	.657
Accuracy (sin^−1^)	1.276753	1.255471	.504/13	.622
Two Back Working Memory Task				
Reaction Time (log)	3.079240	3.067496	.408/13	.690
Accuracy (sin^−1^)	1,146,574	1.148721	−.050/13	.961
Social-Emotional Cognition Task				
Reaction Time (log)	3.426776	3.526582	−1.786/13	.097
Accuracy (sin^−1^)	.881219	1.029940	−2.196/13	.047
Continuous Paired Associate Learning Task				
Time Spent (sec)	268,342.43	245,706.86	1.711/13	.111
Reaction Time (log)	3.426164	3.370106	1.784/13	.098
Accuracy (sin^−1^)	.747632	.851417	−2.749/13	.017
Shopping List Task-Delayed Recall				
Time Spent (sec)	50,757.79	58,700.93	−1.327/13	.207
Accuracy (sin^−1^)	.829577	.866724	−.509/13	.619

### Subjective experiences with tDCS

In general, tDCS was regarded as ‘an electrical treatment to stimulate the brain’, with the purpose to ‘reset your emotion center’ and ‘achieve a balance of the left and right brain’. Regarding adverse effects, participants reported headache (*n* = 3), fatigue (2), dizziness (2), increased emotional reactivity (2), nausea (1), insomnia (1), and pain over scalp contacting electrodes (1). Most adverse effects were tolerable, with reduced intensity after the first 3 sessions.

For depressive symptoms, tDCS responders described universally that depressed mood was ‘much better’, ‘with reduced duration and intensity’. Positive affect was experienced that participants were ‘feeling relaxed’, and ‘able to smile again’. Their motivation increased, energy restored, and the ability to complete tasks resumed. Interestingly, in non-responders, 6 out of 12 participants still reported some improvement in depressed mood; 2 with less anxiety; 2 with attenuated suicide idea, 2 with restored executive function, and 1 with improved appetite. No participants suffered from a deterioration of depressive symptoms. Moreover, we used YMRS to measure manic symptoms in bipolar patients; none of them exceeded a score of 7.

Regarding perceived cognitive effects, 5 participants had increased attention. Memory improved in 4 patients but worsened in 2. Processing speed improved in 1 but deteriorated in 2. The majority of responses were that domains of cognitive ability were unchanged after tDCS treatment.

As for treatment preference, 11 participants listed tDCS as their first choice, reasoning that ‘effects of tDCS were faster and more natural than drugs, with fewer adverse events’. Two participants identified medications as their first choice because of its convenience and immediate effect. Three participants preferred psychotherapy because it helped ‘develop coping skills’ and ‘deal with underlying causes’. Notably, 5 participants had never received psychotherapy.

Overall, tDCS was perceived as safe and tolerable, with substantial effects on depression and equivocal benefits on cognitive ability. It is preferred than pharmacotherapy and psychotherapy in the majority of patients.

## Discussion

The present study demonstrated that tDCS could be advantageous to patients with treatment-resistant depression (TRD) in improving depressive symptoms and cognitive performance. Its effect may be delayed, with marked discrepancy between responders and non-responders. TDCS is well accepted and preferred than other treatment modalities.

The findings here need to be interpreted with a number of limitations considered, including a relatively small sample size, an open-label design, lacking of a controlled group, and less stringent definition of TRD. It is likely that response rates may increase in open-label studies due to positive expectations from both un-blinded patients and un-blinded raters. With the multiple tests for cognitive performance, the 2 significant outcomes may be prone to type-I errors. Also, the inclusion of bipolar patients is likely to increase heterogeneity. Prior studies examining the efficacy of tDCS on TRD were universally small (i.e. sample size < 25), yielding conflictual results. Our positive findings are in agreement with those of Dell’Osso et al. and Ferrucci et al., both applying augmented, F3-F4 tDCS with a five-days, twice-daily protocol [29, 30]. In contrast, in three controlled studies, reduction of depressive symptoms was similar in active and sham groups [26–28]. Results from another open-label study also showed no benefit of tDCS in patients with TRD [34]. The montages of tDCS in these negative studies are mostly different from the established F3-F4 positioning, and their period of observation is, at most, 4 weeks. Comparatively, our tDCS application spans for 6 weeks, with the lengthiest follow-up of 8 weeks, which is more likely to capture its full effect.

Due to the differential antidepressant effect of tDCS, it raises the need to investigate predictors of treatment response. It has been shown that pre-treatment verbal fluency predicts the response of tDCS on depression [35]. In a recent report pooled from 3 tDCS trials with 171 depressed patients, pre-treatment cognitive disturbance, retardation, anxiety and somatization played a role in prediction of response to tDCS [36]. Nonetheless, in our study, no demographic, clinical or cognitive characteristics at baseline could predict response to tDCS, potentially owing to limited sample size, higher severity of depression and unmeasured covariates.

We observed an improvement of visual learning and social cognition at the end of 8 weeks. The result is in accordance with a report by Boggio et al., that, in depressed patients, tDCS stimulation of left DLPFC had a significant effect on improving the accuracy of identifying figures with positive emotional content [37]. Wolkenstein et al. [38] and Brunoni et al. [39] reasoned that tDCS may improve cognition via modifying the emotional inhibitory control and negative attentional bias. Effects of tDCS may extent to cortico-subcortical regions, which can modify the cognitive dysfunction and emotion processing. In contrast, several studies found no effect of tDCS on cognition in patients with TRD [26, 28, 30]. One recent systematic review could not conclude the cognitive benefits of tDCS in depressed patients [40]. Future research is needed to clarify the equivocal findings.

We observed a delayed effect of tDCS over depressive symptoms. In an animal study, cathodal stimulation combined with task assignment showed effects 3 weeks later [41]. In another controlled study examining effects of tDCS in MDD, antidepressant effect in the 3-week masked face was only modest, but the number of responders in the following 3-week, open-label phase was much more encouraging [22]. The after-effects of tDCS have been linked to non-synaptic mechanisms involving neurogenesis [42–44]. TDCS may also induce long-term cortical plastic change via metabolic pathways, for example, increasing BDNF release [41, 45, 46].

## Conclusions

Given that available treatments of TRD had unsatisfactory efficacy or tolerability [9], the high acceptance, perceived benefits, and preference of tDCS demonstrated here have important clinical implications. TDCS is inexpensive and easily administered, which has a potential to serve as a scalable treatment. Our preliminary findings suggest that tDCS can be a desirable option for TRD, however, its efficacy may be delayed; identifying predictors of therapeutic response may achieve more targeted application. Larger controlled studies with optimized montages and sufficient periods of observation are warranted.

## Abbreviations

BDI: Beck Depression Inventory
DSM-5: Diagnostic and Statistical Manual of Mental Disorders-5
ECT: Electroconvulsive Therapy
MADRS: Montgomery–Åsberg Depression Rating Scale; MDD: Major Depressive Disorder
RTMS: Repetitive Transcranial Magnetic Stimulation
TDCS: Transcranial Direct Current Stimulation
TRD: Treatment-Resistant Depression
